# Effects of culinary spices and psychological stress on postprandial lipemia and lipase activity: results of a randomized crossover study and *in vitro* experiments

**DOI:** 10.1186/s12967-014-0360-5

**Published:** 2015-01-16

**Authors:** Cindy E McCrea, Sheila G West, Penny M Kris-Etherton, Joshua D Lambert, Trent L Gaugler, Danette L Teeter, Katherine A Sauder, Yeyi Gu, Shannon L Glisan, Ann C Skulas-Ray

**Affiliations:** Department of Biobehavioral Health, The Pennsylvania State University, 219 Biobehavioral Health Building, University Park, PA 16802 USA; Department of Nutritional Sciences, The Pennsylvania State University, 110 Chandlee Lab, University Park, PA 16802 USA; Department of Food Science, Center for Molecular Toxicology and Carcinogenesis, The Pennsylvania State University, 332 Food Science Building, University Park, PA 16802 USA; Department of Mathematics, Lafayette College, 225A Pardee Hall, Easton, PA 18042 USA; Department of Food Science, The Pennsylvania State University, 332 Food Science Building, University Park, PA 16802 USA

**Keywords:** Spice, Cinnamon, Turmeric, Black pepper, Postprandial lipemia, Pancreatic lipase, Secreted phospholipase A_2_, Psychological stress, TRIER, Postprandial metabolism

## Abstract

**Background:**

Data suggest that culinary spices are a potent, low-calorie modality for improving physiological responses to high fat meals. In a pilot study (N = 6 healthy adults), we showed that a meal containing a high antioxidant spice blend attenuated postprandial lipemia by 30% compared to a low spice meal. Our goal was to confirm this effect in a larger sample and to consider the influence of acute psychological stress on fat metabolism. Further, we used *in vitro* methods to evaluate the inhibitory effect of spices on digestive enzymes.

**Methods:**

In a 2 x 2, randomized, 4-period crossover design, we compared the effects of 14.5 g spices (black pepper, cinnamon, cloves, garlic, ginger, oregano, paprika, rosemary, and turmeric) vs. placebo incorporated into a high fat meal (1000 kcal, 45 g fat), followed by psychological stress (Trier Social Stress Test) vs. rest on postprandial metabolism in 20 healthy but overweight adults. Blood was sampled at baseline and at 105, 140, 180, and 210 minutes for analysis of triglycerides, glucose, and insulin. Additional *in vitro* analyses examined the effect of the spice blend and constituent spices on the activity of pancreatic lipase (PL) and secreted phospholipase A_2_ (PLA_2_). Mixed models were used to model the effects of spices and stress (SAS v9.3).

**Results:**

Serum triglycerides, glucose and insulin were elevated following the meal (p < 0.01). Spices reduced post-meal triglycerides by 31% when the meal was followed by the rest condition (p = 0.048), but this effect was not present during stress. There was no effect of the spice blend on glucose or insulin; however, acute stress significantly increased both of these measures (p < 0.01; mean increase of 47% and 19%, respectively). The spice blend and several of the individual spices dose-dependently inhibited PL and PLA_2_ activity *in vitro*.

**Conclusions:**

Inclusion of spices may attenuate postprandial lipemia via inhibition of PL and PLA_2_. However, the impact of psychological stress negates any influence of the spice blend on triglycerides, and further, increases blood glucose and insulin.

**Trial registration:**

ClinicalTrials.gov as NCT00954902.

**Electronic supplementary material:**

The online version of this article (doi:10.1186/s12967-014-0360-5) contains supplementary material, which is available to authorized users.

## Background

Exaggerated postprandial elevations in triglycerides (ppTG) and glucose (ppG) increase risk of CVD [1-3]. In fact, ppTG concentrations (measured 4 hours post meal) are more strongly correlated with carotid intima-media thickness than fasting triglycerides or low density lipoprotein cholesterol (LDL-C) alone [4]. Elevated ppTG and ppG responses promote atherogenesis, in part, via increases in inflammation, oxidative stress, endothelial dysfunction, and the production of small dense LDL-C[5-10]. For this reason, dietary interventions that attenuate post meal spikes in triglycerides and glucose are clinically important for reducing CVD risk.

There is growing evidence that food-derived polyphenols (from cocoa, red wine, tea, and culinary spices) are protective against chronic disease [11-14]. In a pilot study (n = 6), we showed that adding a 14 g blend of commonly used spices (black pepper, cinnamon, cloves, garlic, ginger, oregano, paprika, rosemary, and turmeric) to a high fat, high carbohydrate meal reduced ppTG concentrations by 31% and insulin by 21% [15]. This finding is especially interesting given that spices provide very few calories and are relatively easy to incorporate into common foods. However, replication of this finding is important, given the small sample size of the pilot study. In addition, a recent study testing a similar spice blend showed no effect on ppTG in adults with type 2 diabetes [16]. The present study was designed to assess the efficacy of a culinary spice blend to blunt ppTG following a high fat meal in healthy, but overweight/obese adults.

We also considered the contribution of acute psychological stress to the metabolism of a high fat meal, in the presence and absence of spices. We have previously shown that exposure to brief psychological stressors, presented under controlled, laboratory conditions, blunts the clearance of triglycerides [17]. Others have found that acute stress increases blood glucose [18,19], likely a function of enhanced insulin resistance [20] and that greater number of prior day stressors is associated with reduced post meal fat oxidation [21]. We addressed these aims using a 2 x 2 design in which high fat, high carbohydrate meals were presented with and without spices, and in the presence and absence of laboratory stress tasks.

Finally, we conducted *in vitro* studies to explore digestive enzyme inhibition as a potential mechanism of action for the spice blend. Dietary polyphenols are found in the blood only in trace amounts [22], and there is growing evidence that their beneficial effects on postprandial metabolism take place in the gut [23-25]. Thus, we tested whether the spice blend, or its component spices, had a measurable, inhibitory effect against enzymes critical for fat digestion in the small intestine (pancreatic lipase [PL] and phospholipase A_2_ [PLA_2_]).

## Methods

### Design

We conducted a randomized, controlled, 4-period crossover study with at least one week separating testing sessions. Participants were randomized to the following conditions, presented in counterbalanced order: 1) spice meal + rest, 2) spice meal + stress, 3) control meal + rest, and 4) control meal + stress. A computer generated randomization scheme was developed in advance for the four treatment conditions. The randomization scheme used a balanced block size of 4 to ensure even distribution among groups. Eligible participants were assigned to treatments at the baseline visit by the study coordinator. Due to the nature of the treatment conditions (e.g. spice and stress), blinding of the individual collecting blood samples was not possible. Statistical analyses were conducted without knowledge of treatment assignment for each individual. Participants were not informed in advance of the visit whether they would have a “stress” or “rest” visit, and identical environmental conditions were used prior to the stress/rest period. Samples were labeled only with subject id, period, and time point identifiers, such that outcomes assessment was blinded. Premenopausal women (n = 2) were scheduled during the first 7 days of the menstrual cycle. The protocol was approved by the Institutional Review Board of The Pennsylvania State University and written informed consent was obtained from all participants.

### Participants

Twenty healthy but overweight or obese men and women completed this study (n = 6 women). Inclusion was limited to those who were aged 30–65 y, free from any serious illness (including any inflammatory conditions, liver or kidney dysfunction, a history of heart disease), had body mass index of 25–40 kg/m^2^, resting blood pressure (BP) < 160/100 mmHg (if BP was ≥140/90, approval for study participation was requested from the participant’s physician), fasting glucose <126 mg/dL, and willingness to discontinue all dietary supplements during the study. Additionally, potential participants were excluded if they used tobacco products, were training for athletic competition (>2 h aerobic activity a week), or used medications relating to birth control, hormone replacement therapy, lipid lowering, BP lowering, and psychosis or depression, with the exception of selective serotonin reuptake inhibitors.

Interested individuals (n = 118) were screened by phone after answering advertisements from community bulletin boards and email lists. Thirty-one met the initial criteria and were scheduled for a clinic screening; 2 cancelled their visits therefore only 29 were screened in the clinic. At the screening visit, inclusion criteria were checked via blood sampling for a complete blood count and chemistry panel, measurements of weight and height, and BP assessment according to JNC 7 guidelines [26]. Twenty-four individuals passed the clinic screening and were approved to participate but only 22 started the study. Two participants were withdrawn during the study due to exceeding limits for BP (n = 1) and emotional response to the stress task (n = 1). Thus, data are reported for 20 healthy participants, including 6 women.

### Procedures

**Figure 1:**
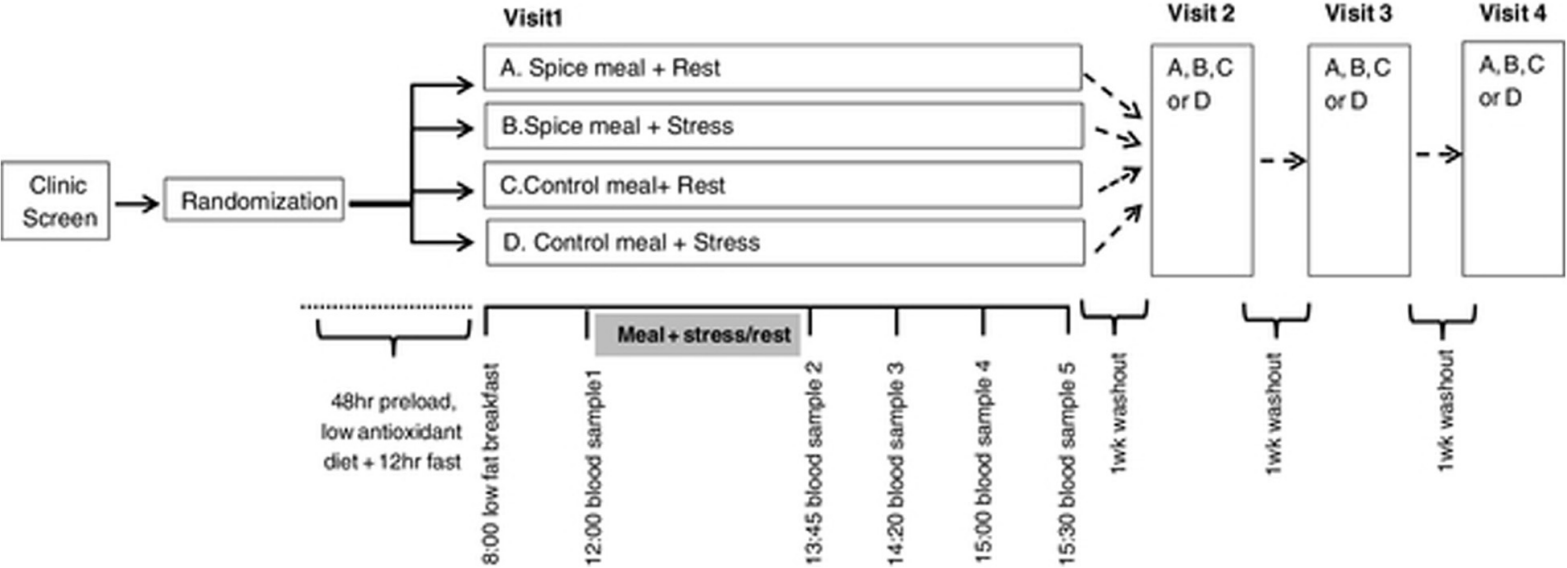
**Study design and timeline on testing days.**

In the 48 hours prior to each testing session, participants consumed one meal per day (provided) which matched their treatment assignment for the laboratory session (e.g. a meal containing spices was consumed vs. a meal without spices + placebo capsules). Because of the distinctive flavors in the spice meal, true blinding of the participants was not possible. Instead, participants were told that the purpose of the study was to compare the effects of antioxidants provided in meals vs. antioxidants provided in capsules. The placebo capsules provided with the control meal contained only crystalline methylcellulose, a commonly used placebo. Participants were asked to avoid foods containing antioxidants in the 48 hours prior to visits, and a list of such foods was provided. Participants self-reported compliance with food restrictions on the morning of each visit. Participants also fasted overnight and consumed a standardized, low antioxidant breakfast (white bagel and nonfat spread were provided) approximately 3 hours prior to arrival at the clinic and 4 hours prior to the baseline blood draw and test meal. Following intravenous (IV) catheter insertion and the baseline blood sample, participants were given 30 minutes in which to consume the test meal followed by a stress or rest condition. The second blood sample was collected via the IV catheter approximately 75 minutes after the test meal was completed (105 minutes after the fasted baseline blood draw). Additional blood samples were collected at 140, 180 and 210 minutes after the baseline blood draw. A schematic of testing day procedures is provided in Figure 1. Recruitment and data collection were active between May 2009 and May 2011. All testing was completed at the clinical research center on the campus of The Pennsylvania State University (University Park, PA).

### Test meal

The 1000 kcal lunch meal consisted of a chicken and white rice dish, a corn muffin, and pastry dessert. The test meal was designed to include large doses of fat (~43 g) and carbohydrates (~98 g) which are known to cause substantial postprandial changes in glucose, insulin and triglycerides [27,28]. Additional specifics about the fatty acid composition of the meal can be viewed in the Additional file 1: Table S1. The control meal was prepared without spices, and placebo capsules were consumed with the meal. The spice blend (14.5 g/meal) was incorporated into the various meal items (see Table 1). The spices were donated by the Characterized Samples Program at the McCormick Science Institute (Hunt Valley, MD). Spices were weighed with a balance accurate to 0.01 g (Mettler Toledo, Columbus, OH). The palatability of this blend was previously tested [15]. In spite of the relatively high dose of spices, participants reported no gastrointestinal side effects and all were able to consume the meal within the allotted time period.

**Table 1:** **Characteristics of the spice blend that was added to create the spiced meal**

**Spice**	**Dessert biscuit,** ***g***	**Coconut chicken,** ***g***	**Cheese bread,** ***g***	**Total dose,** ***g***	**H-ORAC contribution,** ^**1**^ ***μmol TE***	**L-ORAC contribution,** ^**1**^ ***μmol TE***	**Phenolic contribution,** ^**1**^ ***mg GAE***
Black pepper		0.45	0.45	0.91	93	212	3
Cinnamon	0.88	0.23		1.11	1590	37	50
Cloves	0.30	0.31		0.61	680	1091	101
Garlic powder		0.91	0.90	1.81	118	3	1
Ginger	0.38	0.75		1.51	138	451	10
Oregano (Mediterranean)		1.13	1.13	2.26	3745	510	86
Paprika		1.43	1.42	2.85	392	233	47
Rosemary			0.61	0.61	684	324	30
Turmeric		2.09	0.70	2.79	1249	2296	77
Total				14.5	8690	5157	404

^1^ORAC values (*in vitro*) are from the USDA 2010 Report [29]. Abbreviations: H-ORAC, hydrophilic ORAC; L-ORAC, lipophilic ORAC. Contribution of ORAC and phenolic compounds was calculated by weight using the values reported in the table.

### Stress task

The Trier Social Stress Test, a standardized and scripted procedure for producing reliable increases in cortisol, BP and heart rate (HR), has been described in detail elsewhere [30]. In brief, subjects were asked to prepare (10 min) and deliver (5 min) a speech and to complete serial subtraction (8 min) while in the presence of “expert panelists” who provided no encouragement and offered negative comments on the person’s performance. Subjects listened to gentle music during a 20 min rest period which preceded the stressors and a 10 min recovery which followed them. To prevent habituation, the topic for the speech task and the numbers for the serial subtraction were changed for the second stress visit. BP and HR were monitored repeatedly throughout each task using a standard BP cuff (Dinamap Pro 100 oscillometric monitor, GE Medical Systems).

### Biochemical analysis

Whole blood was transferred into serum separator tubes, allowed to clot, and centrifuged. Total cholesterol and triglycerides were determined by enzymatic procedures (Quest Diagnostics, Pittsburgh, PA; CV < 2% for both). HDL-C was estimated according to the modified heparin-manganese procedure (CV < 2%). LDL-C was not interpreted because calculated LDL-C is not accurate in the presence of ppTG. Insulin was measured by radioimmunoassay using ^125^I-labeled human insulin and a human insulin antiserum (Quest Diagnostics). Glucose was determined by an immobilized enzyme biosensor using the YSI 2300 STAT Plus Glucose & Lactate Analyzer (Yellow Springs Instruments, Yellow Springs, OH).

### Digestive enzyme inhibition by spices in vitro

Extracts of the entire spice blend and each individual spice were prepared by extracting spices with 10 volumes of acetone:water:acetic acid (80:20:0.1%, v:v:v) overnight. The organic solvent was removed under vacuum and the remaining aqueous phase was freeze-dried. Lipase from porcine pancreas (PL, type II) and 4-nitrophenyl butyrate (4-NPB, 98%) were purchased from Sigma-Aldrich (St. Louis, MO). Stock solutions were prepared in dimethylsulfoxide (EMD Chemicals Inc.) and stored at −20°C. An EnzChek Phospholipase A_2_ (PLA_2_) Assay Kit was purchased from Invitrogen (Carlsbad, CA). All other reagents were of the highest grade commercially available.

Inhibition of PL by the spice blend and individual spice extracts was tested by monitoring the cleavage of 4-NPB to release 4-nitrophenol. PL was suspended in water (10 mg/mL) and incubated at 37°C for 5 min. The solution was centrifuged for 5 min at 664 x *g* and the supernatant was then used as the enzyme source for subsequent experiments. For each experiment, the PL supernatant was diluted 1:50 in buffer solution (20 mM Tris–HCl, 1.3 mM CaCl_2_, 150 mM NaCl, pH = 8.0). Spice extracts (0–200 μg/mL) were combined with PL and 4-NPB (0.2 mM) was added to start the reaction. Following incubation at 37°C for 10 min, absorbance was read at 400 nm. Inhibition of PLA_2_ was examined using a commercially available fluorometric method (Invitrogen). Buffered PLA_2_ solution (1 U/mL, pH 8.9) and spice extracts (0–200 μg/mL) were combined in a 96-well plate. A fluorogenic PLA_2_ substrate (Red/Green BODIPY PC-A2, 1.67 μM) was dispensed to each well to start the reaction. After incubation at room temperature in the dark for 10 min, fluorescence was determined at λ_ex_ = 485 nm and λ_em_ = 538 nm (Fluoroskan Ascent FL, ThermoFisher Scientific Inc.).

### Statistical analysis

Based on our pilot data, a sample size of 10 was necessary to observe a difference of 21 units in the postprandial change in triglycerides between treatment and control (power = 0.90, α = 0.05). However, a sample size of 20 was recruited to provide additional power to observe differences between the stress and rest conditions. The mixed models procedure (PROC MIXED, SAS v9.3, Cary, NC) was used to test the fixed effects of treatment (spice or placebo), condition (stress or rest testing session), task (baseline, speech preparation, speech task, math and recovery) and their interaction. Metabolic endpoints were analyzed as change from baseline (value minus baseline value) with doubly repeated measures models using unstructured and compound symmetric covariance structures for time points and visits, respectively. BP and HR were modeled with subject treated as a random effect. Data were tested for normality and glucose was transformed by square root to achieve normality. Least-squares means and standard errors are reported. Model selection was based on optimizing fit statistics (evaluated as lowest Bayesian Information Criterion). Baseline values were included as covariates in these models. The last three hemodynamic measurements of the resting and recovery periods were averaged. For the speech preparation, speech delivery, and math tasks, measurements were taken every minute and averaged by task. Stress analysis was completed for N = 19, as one participant found the procedure to be too aversive. For *in vitro* inhibition experiments, the half maximal inhibitory concentration (IC_50_) was determined as the extract concentration which inhibited enzyme activity by 50%.

## Results

Participants were normotensive and free of chronic disease, although they were overweight/obese and had moderate hyperlipidemia (Table 2). Baseline glucose, insulin, triglycerides, BP, and HR were not different on the mornings of spice and control visits (Table 3). This finding indicates that the 48 hour “preload” period (during which meals--with and without spices--were consumed in the 48 hours prior to the testing session) had no effect on fasting endpoints.

**Table 2:** **Participant characteristics ascertained at fasted screening visit**

**Characteristic**	**Mean ± SEM**
Age (years)	43.2 ± 2.3
Body mass index (kg/m^2^)	30.4 ± 0.9
Systolic blood pressure (mmHg)	117.8 ± 2.2
Diastolic blood pressure (mmHg)	81.8 ± 1.3
Triglycerides (mmol/L)	1.54 ± 0.14
Glucose (mmol/L)	5.23 ± 0.12
Total cholesterol (mmol/L)	4.78 ± 0.30
HDL-C (mmol/L)	1.18 ± 0.05
LDL-C (mmol/L)	2.89 ± 0.13
Total to HDL ratio	4.2 ± 0.22
C-reactive protein (mg/L)	12.95 ± 2.00

**Table 3:** **Baseline blood sample values at placebo and spice testing visits following 48 hr preload**

**Characteristic**	**Control visits**	**Spice visits**	***p***
Systolic blood pressure (mmHg)	121.60 ± 1.83	119.5 ± 2.41	0.23
Diastolic blood pressure (mmHg)	80.80 ± 1.22	80.25 ± 1.62	0.70
Heart rate (bpm)	64.43 ± 1.56	64.98 ± 1.84	0.56
Triglycerides (mmol/L)	1.76 ± 0.13	1.82 ± 0.49	0.51
Glucose (mmol/L)	5.00 ± 0.15	5.01 ±0.18	0.28
Insulin (pmol/L)	63.00 ± 9.69	60.56 ± 9.11	0.77

Note: Values obtained at baseline of each test day, approximately 4 hours after the standardized breakfast.

### Plasma metabolites

**Figure 2:**
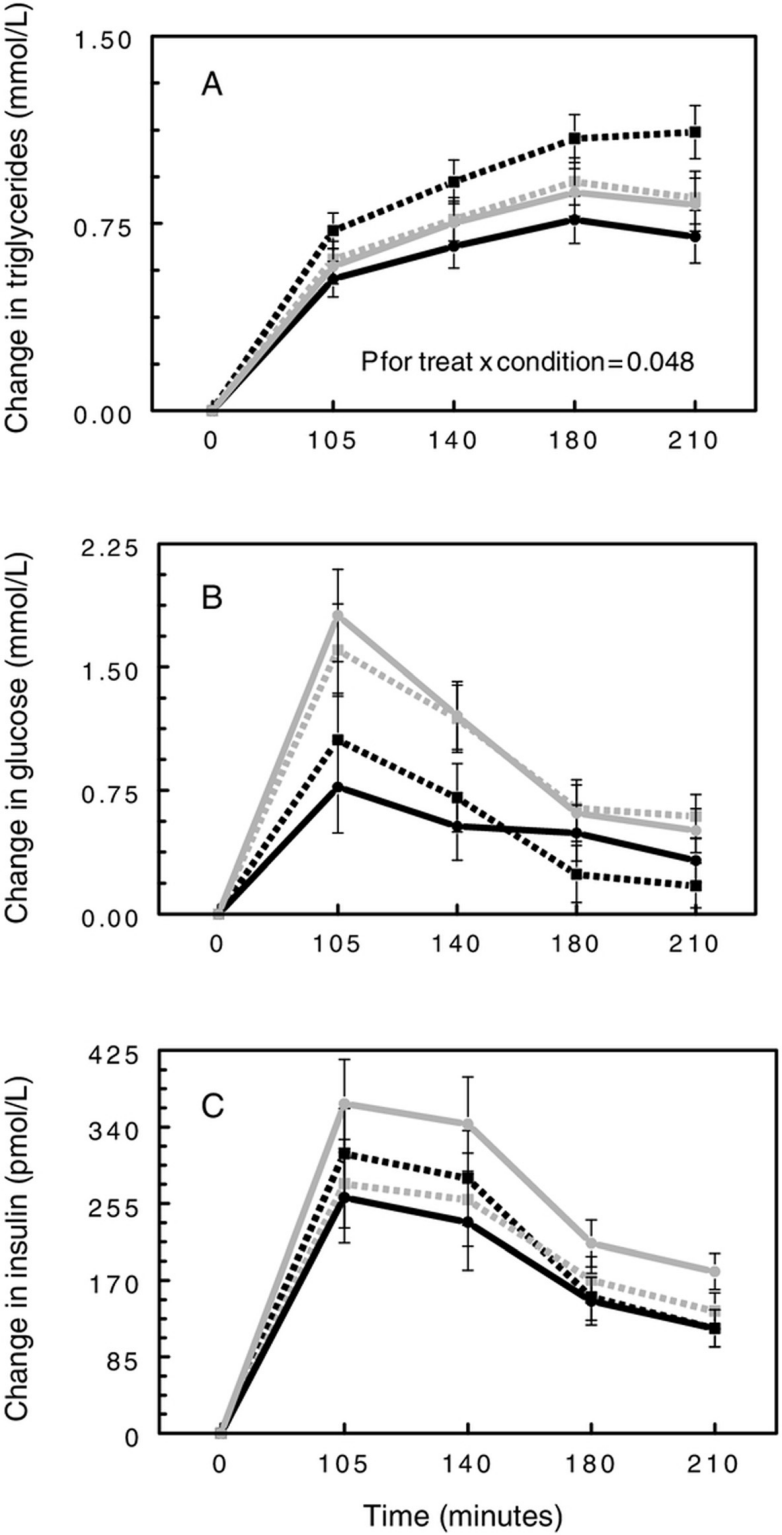
**Change in triglycerides (A), glucose (B) and insulin (C) over the course of the study visit by treatment and condition: placebo rest (**  **) , placebo stress (**  **), spice rest (**  **) and spice stress (**  **).**

Plasma concentrations of insulin, glucose, and triglycerides following consumption of the high fat meal for each combination of treatment (spice or placebo) and condition (stress or rest) are shown in Figure 2. Analysis of the change in post meal triglycerides revealed a main effect of time (p < 0.01, indicating that meal consumption increased TG). However, there was a treatment x condition interaction (p = 0.048) as well. Review of *post hoc* tests showed that we replicated the finding from our previous study: including spices in the high fat meal significantly attenuated the ppTG response (mean change of 0.66 ± 0.08 vs. 0.96 ± 0.08 mmol/L, respectively, Tukey p = 0.013). However, this pattern was evident only when the participants were allowed to rest during the postmeal period. When the spice meal was combined with stressor exposure, attenuation of ppTG was not observed.

**Figure 3:**
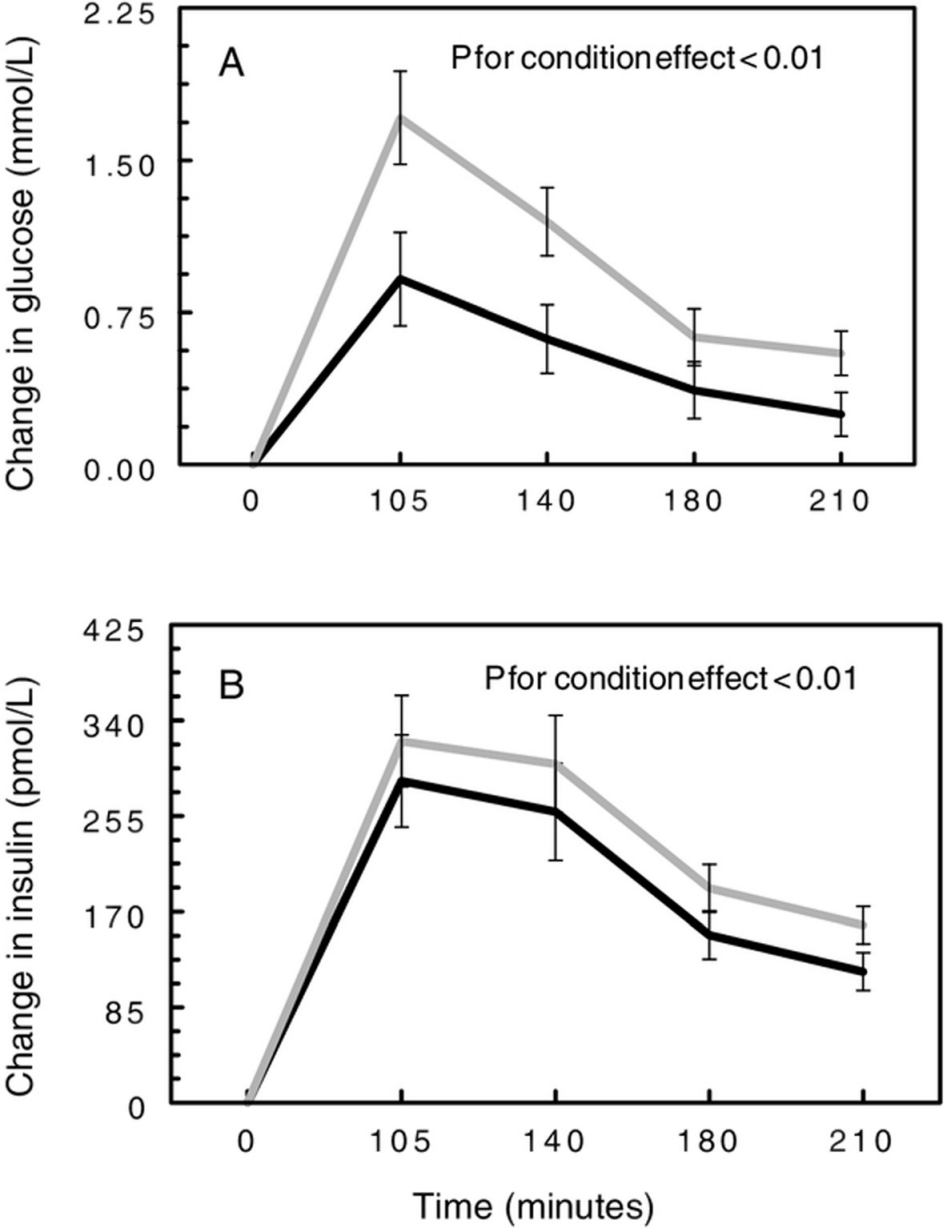
**Change in glucose (A) and insulin (B) by main effect of condition: stress (**  **) and rest (**  **).**

As expected, main effects of time were present for both insulin and glucose (p <0.01), with peaks observed at 105 and 140 minutes (Figures 2 and 3). No treatment effect was observed for insulin. However, a significant main effect of condition was present (p < 0.01; Figure 3), such that the stress condition resulted in higher insulin levels than the rest condition regardless of treatment or time (mean change of 234.74 ± 22.22 vs. 196.54 ± 22.91 pmol/L, respectively). A similar pattern was observed for glucose (p < 0.01): glucose concentrations were higher after the stress condition compared to the rest condition (mean overall change of 1.02. ± 0.11 vs. 0.53 ± 0.12 mmol/L, respectively; Figure 3). No changes in glucose or insulin could be attributed to the addition of spices.

### Hemodynamics in response to stress

**Figure 4:**
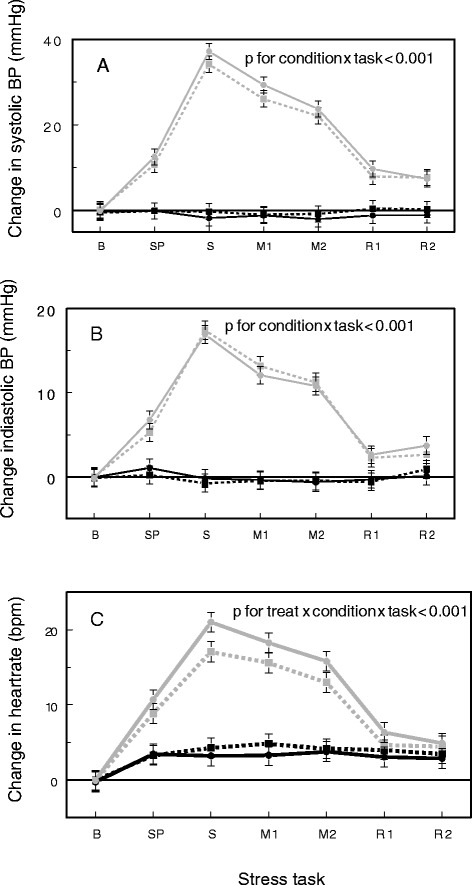
**Change in systolic blood pressure (A), diastolic blood pressure (C) and heart rate (C) by treatment and condition: placebo rest (**  **) , placebo stress (**  **), spice rest (**  **) and spice stress (**  **).** Tasks are as follows: **B** (baseline), SP (speech preparation), S (speech), M1 (math 1), M2 (math 2), R1 (recovery 1) and R2 (recovery 2).

Both diastolic and systolic BP and heart rate (HR) increased significantly in response to the stress task (Figure 4). Although the stressors significantly increased BP compared to the resting visits and the resting baseline (condition x task interaction p < 0.001, *post hoc* Tukey p ≤ 0.01), the addition of spices to the meal had no impact on BP response to stress. In contrast, there was a significant interaction of treatment x condition x task (p < 0.001) on HR. Spices and stress had additive effects on heart rate; higher HR was observed during the speech task and during both math tasks when the high fat meal included spices (*post hoc* ps ≤ 0.0035).

### Digestive enzyme inhibition by spices in vitro

**Figure 5:**
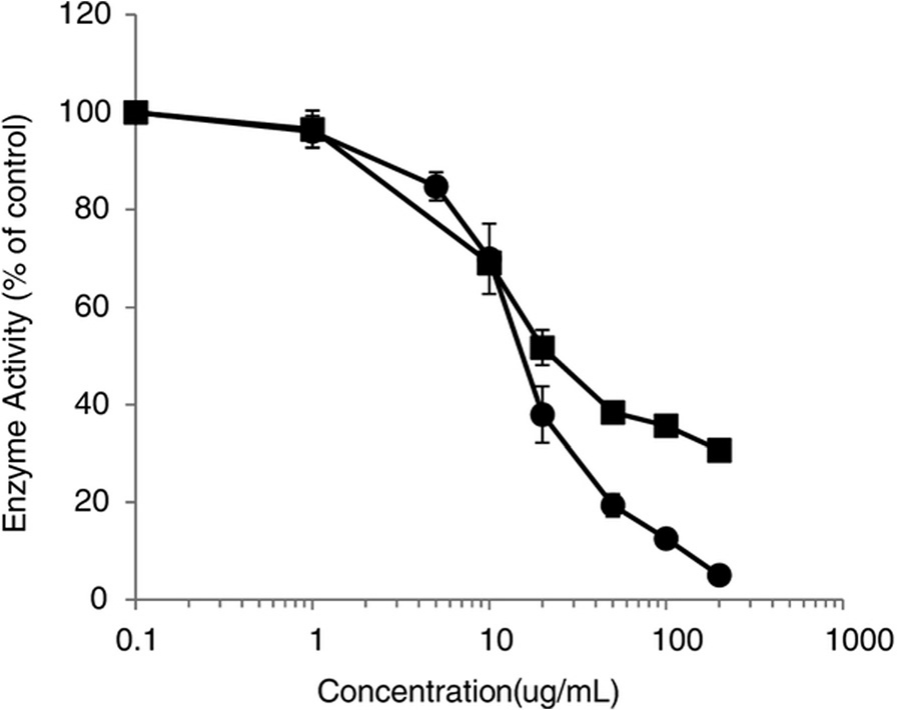
**Inhibition of secreted pancreatic lipase (**  **) and phospholipase A**_**2**_ **(**  **)** ***in vitro*** **by the spice blend.** Values are normalized to vehicle-treated controls and expressed as the mean ± SD of at least three independent experiments.

The spice blend extract dose-dependently inhibited both PL and PLA_2_ (Figure 5). Although the spice blend had similar IC_50_ values for PL (17.2 μg/mL) and PLA_2_ (23.6 μg/mL), it had greater inhibitor efficacy against PLA_2_: greater than 90% inhibition at the highest concentration tested compared to 70% inhibition of PL.

**Figure 6:**
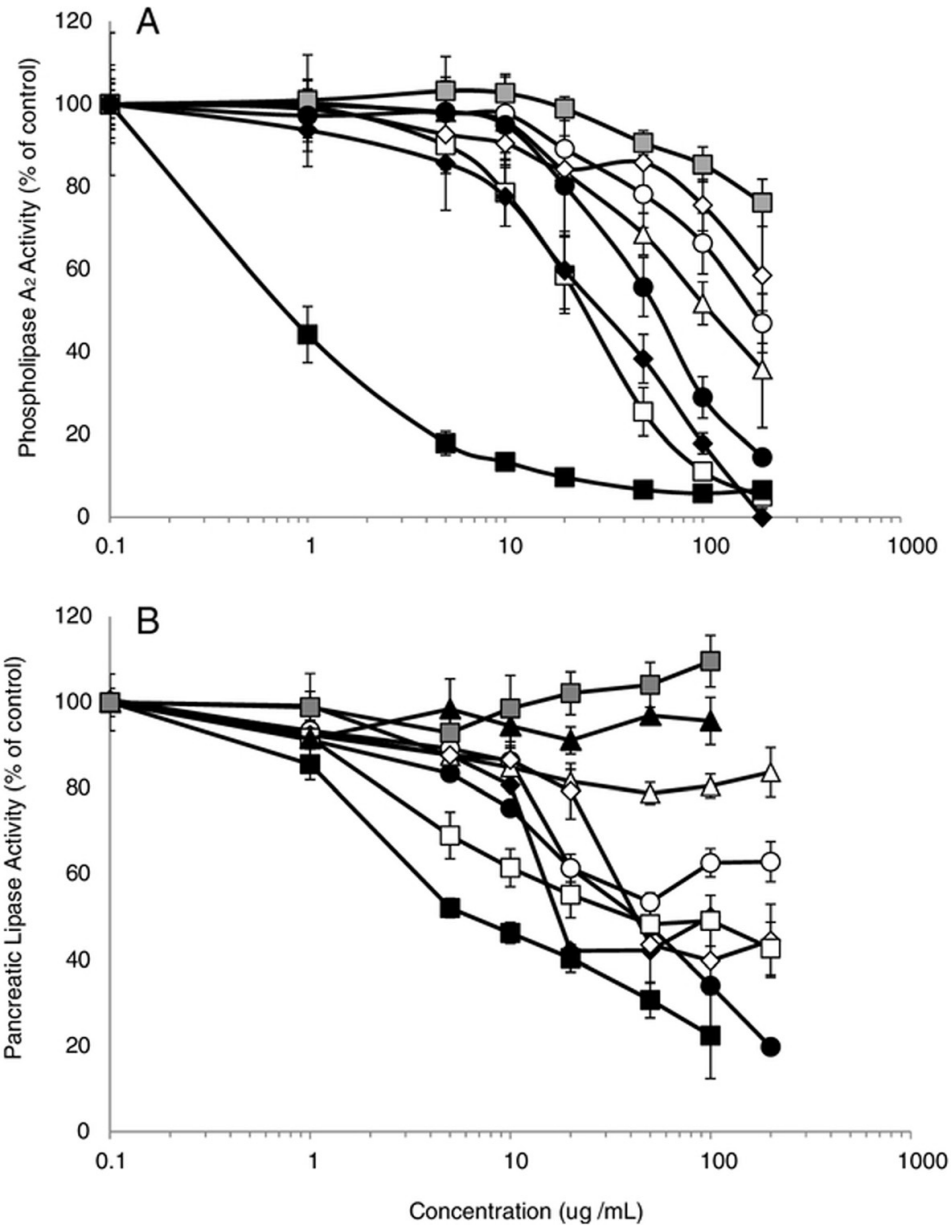
**Inhibition of phospholipase A**_**2**_ **(A) and pancreatic lipase (B) activity by individual spice extracts: black pepper (**  **), clove (**  **), cinnamon (**  **), ginger (**  **), oregano (**  **), paprika (**  **), rosemary (**  **), garlic (**  **) and turmeric (**  **).** Values are normalized to vehicle-treated controls and expressed as the mean ± SD of at least three independent experiments. Due to color interference with the PL assay, values could not be obtained for turmeric or cinnamon at 200 μg/ml. Interference with the PLA_2_ assay prevented values from being obtained for garlic.

The inhibitory effects of extracts of the component spices against PL and PLA_2_ were compared (Figure 6). Cinnamon was the most potent inhibitor of PLA_2_ (IC_50_ = 7.9 μg/mL), followed in potency by cloves (IC_50_ = 26.5 μg/mL) and turmeric (IC_50_ = 31.4 μg/mL). Paprika and oregano were the least active spices and inhibited PLA_2_ by 23.9 and 41.4%, respectively, at the highest concentration tested (200 μg/mL). Similarly, cinnamon (IC_50_ = 5.5 μg/mL), turmeric (IC_50_ = 18 μg/mL), and cloves (IC_50_ = 38 μg/mL) were the most potent inhibitors of PL. Black pepper, garlic, and paprika were the least potent inhibitors of PL, with paprika and garlic failing to show any inhibitory activity.

## Discussion

In this study of otherwise healthy overweight/obese adults, we confirmed that adding a blend of commonly used culinary spices to a high fat meal significantly reduced the postprandial serum triglyceride response by approximately 31%. However, this pattern was only evident when the postmeal period involved an extended rest period. When participants engaged in a 25 min long battery of performance-based, standardized, stressor tasks during the postmeal period, this beneficial effect of spices was not evident. We have previously demonstrated the triglyceride lowering effect of spices in a small pilot study [15], while others found no effect [16]. This latter, null finding was likely due to the minor postprandial change in triglycerides induced by the test meal that contained ~25 g of fat. The 43 and 50 g fat meal challenges used in our protocols provided a magnitude of change large enough for differences to be ascertained. An ~50 g fat dose is recommended for clinical trials evaluating ppTG [27], and it is ecologically valid, considering the pervasiveness of high fat meals in Western diets. Notably, we did not replicate an earlier finding from our pilot study (n = 6, males only), in which a very similar spice blend attenuated insulin levels by ~21% in the postprandial period.

To explore the mechanism for ppTG lowering, we examined the inhibitory effects of the spice blend and the component spices against two intestinal digestive enzymes that are important for fat metabolism, pancreatic lipase (PL) and phospholipase A_2_ (PLA_2_). In concert, the spices inhibited 50% of enzymatic activity at concentrations of less than or equal to 25 μg/mL. Cinnamon was the most potent inhibitor of both enzymes, inhibiting the activity of both by 50% at concentrations less than 8.0 μg/mL. Turmeric and cloves were also potent inhibitors of both enzymes. These results suggest that inhibition of enzymes required for lipid absorption, resulting in reduced chylomicron release into circulation, may be at least one mechanism by which the spice blend reduced post meal triglycerides.

The polyphenolic constituents of other food products, such as cocoa, have been shown to inhibit lipase activity [25], thus the *in vitro* lipase inhibition produced by the spice blend may be due to its high polyphenol content. The component spices in the blend may also have interacted additively or synergistically *in vivo* to produce the effect observed. For example, although we found that black pepper had little inhibitory activity against PL or PLA_2_*in vitro*, it is known to slow gastric emptying [31]. Thus, black pepper may have prolonged the residence time of the more inhibitory spices (eg. cinnamon, cloves, turmeric) in the upper GI tract and provided prolonged time for inhibition. Alternatively, flavonoid and polyphenolic compounds (large amounts of which are present in the spice blend) have been shown to reduce chylomicron formation in human intestinal cells via reduced intracellular cholesterol synthesis [32,33]. This observation suggests that these compounds may also down regulate chylomicron synthesis independent of enzyme inhibition and fat absorption.

As expected, exposure to acute social stress increased BP and HR. It also increased post meal glucose and insulin and prevented the triglyceride lowering effect of the spices. Although acute psychological stress has long been known to interfere with triglyceride clearance [17] and increase ppTG [34], these data are the first to suggest that acute stress reverses some of the beneficial effects of dietary polyphenols on lipid metabolism. Lipolysis and subsequent triglyceride synthesis are promoted via glucocorticoid receptor binding [35,36], suggesting a mechanism through which stress-induced cortisol release could impact triglyceride response. Future research is required to identify the underlying mechanism(s) that mediate this effect.

Stress induced increases in postprandial glucose have been previously documented [18,19,37], however these are the first data to show this effect in the absence of disorders known to influence either glucose concentrations (diabetes) or stress reactivity (post-traumatic stress disorder). We observed dramatic elevations in glucose (47%) following stress vs rest with lesser increases in insulin response (19%). This finding supports the theory that stress diminishes peripheral tissue sensitivity to insulin, blunting the uptake of glucose [38]. Clinicians should be aware of the influence of stress on glucose levels which could be particularly relevant to diagnostic oral glucose tolerance testing for diabetes mellitus.

It is interesting that HR was higher during the stress tasks (but not at rest or recovery) when the spice meal was consumed. This increase was not anticipated, and given its observation only during the stressful tasks, it is difficult to interpret. Increased sympathetic activity, which results in increased HR, is the major pathway for food induced augmentation of energy expenditure or thermogenesis [39-41]. Like the well-studied capsaicin compound from hot peppers, constituents of ginger, black pepper and garlic (all contained in the spice blend) stimulate catecholamine release from the adrenal medulla in mice [42,43]. Others have postulated a thermogenic effect of pungent spices, and ginger has previously been implicated as a thermogenic augmenter in humans [44], however, not consistently [45]. This study is the first of which we are aware to demonstrate increased sympathetic activity in humans following a blend of these spices.

The 2x2 design of this study provides insight into both the individual effects of stress and spices on postprandial metabolism and also their interactive effect. Unfortunately, true blinding was not possible given the strong flavors and vivid coloring present in the spiced meal and the obvious designation of stress or rest. However, statistical analyses were conducted in a blinded fashion and participants were not informed prior to study visits which conditions they would be experiencing on a given test day. While the sample was reflective of a common phenotype in the United States (overweight but disease free), the study was not powered to explore differences by sex, genetic makeup or lifestyle differences—all of which are known to influence postprandial metabolism on some level [46].

## Conclusions

Post meal triglycerides are an important indicator of cardiovascular risk and a potential target for therapeutic intervention. We have shown that the post meal triglyceride response can be blunted by the inclusion of a culinary spice blend in a high fat meal. Further, we have shown data that support an inhibitory role of spices against enzymes responsible for lipid digestion in the small intestine. These data suggest that the regular inclusion of spices in the diet may help attenuate the effect of large fat loads on cardiovascular risk. However, the impact of psychological stress negates any influence of the spice blend on triglycerides, and further, increases blood glucose and insulin. These findings suggest that stress management may be a more potent intervention for cardiovascular risk management than inclusion of spices. Nevertheless, spices do beneficially affect the postprandial TG response when stress is not an underlying pretense. Finally, the complex interactions between stress and meal type provide further evidence that the frequent daily occurrences of meal times and sympathetic arousal need to be studied in concert.

## Additional file

Additional file 1: Table S1. Macronutrient and fatty acid profile of the control meal.
