# Supplementary material for: Effects of culinary spices and psychological stress on postprandial lipemia and lipase activity: results of a randomized crossover study and in vitro experiments
Source: J Transl Med. 2015 Jan 16;13:7. doi: 10.1186/s12967-014-0360-5 (PMC4322464; doi:10.1186/s12967-014-0360-5)
Supplement: Additional file 1: Table S1. — Macronutrient and fatty acid profile of the control meal. [file 12967_2014_360_MOESM1_ESM.docx]

Additional file 1: Table S1. Macronutrient and fatty acid profile of the control meal

|  | Energy, *Kcal* | Fat, *g* | Carbohydrate, *g* | Protein, *g* | Cholesterol, *mg* | Saturated fatty acids, *g* | Mono-unsaturated fatty acids, *g* | Poly-unsaturated fatty acids, *g* | Fiber, *g* |
| --- | --- | --- | --- | --- | --- | --- | --- | --- | --- |
| Dessert biscuit | 106 | 4.0 | 16.1 | 1.4 | 0 | 1.1 | 1.5 | 1.3 | 0.4 |
| Coconut chicken | 472.3 | 22.7 | 40.4 | 26.6 | 60 | 17.8 | 2.0 | 1.0 | 1.2 |
| Corn cheese muffin | 367 | 15.8 | 41.3 | 14.9 | 68 | 5.7 | 5.7 | 2.7 | 1.3 |
| Total | 945.3 | 42.5 | 97.8 | 42.9 | 128 | 24.6 | 9.2 | 5 | 2.9 |

Supplemental Material

List 1. Foods restricted in the 48hrs prior to testing visits

o   Hot or Cold Tea (1 cup per day of tea or coffee was permitted)

o   Hot or Cold Coffee (1 cup per day of tea or coffee was permitted)

o   Berries/Berry Juice (Including Acai)

o   Grapes/Grape Juice

o   Pomegranate

o   Soy Products (e.g. tofu)

o   Cocoa Powder and Dark Chocolate

o   All Spices (including pepper)

o   Mustard

o   Hot Sauce

o   Processed Tomato Products (except small amounts of ketchup or pizza sauce)

o   Vegetable Juices

o   Dark, Leafy Greens (kale, spinach, etc)

o   Kidney and Black Beans

o   Pecans or large amounts of any other nuts

o   Artichokes
